# End-of-Life Care and Use of Hospital Resources in Radiotherapy-Treated Cancer Patients with Brain Metastases: A Single-Institution Retrospective Study

**DOI:** 10.1089/pmr.2024.0017

**Published:** 2024-08-02

**Authors:** Nelli-Sofia Nåhls, Anu Anttonen, Eliisa Löyttyniemi, Antti Jekunen, Outi Akrén, Tiina Saarto

**Affiliations:** ^1^ Department of Oncology, Vaasa Central Hospital, The Wellbeing Services County of Ostrobothnia, Vaasa, Finland.; ^2^ Department of Oncology, Comprehensive Cancer Centre, University of Helsinki, Helsinki, Finland.; ^3^ Department of Radiotherapy, Comprehensive Cancer Center, Helsinki University Hospital, Helsinki, Finland.; ^4^ Department of Biostatistics, University of Turku and Turku University Hospital Turku, Turku Finland.; ^5^ Department of Oncology, Turku University, Vaasa, Finland.; ^6^ Palliative Center, Tyks Hospital Services, Turku University Hospital, Turku, Finland.; ^7^ Department of General Medicine, University of Turku, Turku, Finland.; ^8^ Department of Palliative Care, Comprehensive Cancer Center, Helsinki University Hospital, and Faculty of Medicine, Helsinki University, Helsinki, Finland.

**Keywords:** brain metastases, emergency department, end of life, hospital services, palliative care decision

## Abstract

**Purpose:**

The aim of this single-institution retrospective study of patients treated with radiotherapy for brain metastases (BM) was to evaluate the timing of the palliative care (PC) decision, the use of health care services, i.e., emergency department (ED) visits and hospitalizations, and the implementation of radiotherapy at the end of life (EOL).

**Methods:**

Data on all cancer patients with BM treated in Finland at the Vaasa Central Hospital Radiotherapy Department between March 2011 and November 2020 were retrospectively reviewed. The follow-up period lasted until November 2021. Altogether, 91 patients (54 men, mean age 67 years [range 23–91 years]) were analyzed. Data on timing of PC decision, visits to the PC outpatient unit, and ED and hospitalization periods were collected retrospectively from patients’ records.

**Results:**

The median overall survival from diagnosis of BM was 3.7 months (range 1–62 months) and, after radiotherapy, 2 months (0–61 months). Thirty-two percent of the patients received radiotherapy in the last month of life. During the last 30 days of life, 44 patients (48%) visited the ED and 38 (42%) were hospitalized. Patients with an early PC decision (>30 days before death) had fewer hospitalizations (22% vs. 53%; *p* = 0.005) and died less often during the hospitalization period (9% vs. 27%; *p* = 0.047) at EOL. No significant difference was found in ED visits (41% vs. 53%; *p* = 0.28).

**Conclusion:**

For a large proportion of patients with BM, the prognosis is very poor. It is important to identify these patients and abstain from radiotherapy at EOL to reducing inappropriate health care utilization.

## Key Message

It is important to identify patients with brain metastases with a poor prognosis so that end of life care can be organized appropriately and acute hospitalization just before death can be avoided.

## Introduction

Secondary malignant lesions in the brain are the most common intracranial tumors in adults, occurring in up to 20%–30% of adults with cancer at some point in their disease trajectory.^1–4^ Lung cancer, breast cancer, and melanoma are the most common tumors causing brain metastases (BM).^5^ BM usually occur at a later point, but BM symptoms are the first sign of metastatic cancer for a certain group of cancer patients.^6^ The choice of treatment varies depending on several factors including, for example, the number of BM and patients’ performance status. In recent years, the treatment of both extracranial disease and BM has improved.^7–9^ The most common treatment options for BM are stereotactic radiation therapy (SRT/SRS), whole brain radiation therapy (WBRT), and systemic therapy. For some patients, surgery is an option, especially if it is needed to confirm the histological diagnosis or for quick relief of symptoms.^2,7^

Despite developments in treatment, BM are a sign of poor prognosis in most of the cases.^10,11^ It is uncertain whether patients with a very poor prognosis and a significant burden of disease will benefit from WBRT at all. The randomized Quality of Life after Treatment for Brain Metastases (QUARTZ) study showed no difference in survival, quality of life (QOL), or use of dexamethasone between WBRT and best supportive care in patients with non-small cell lung cancer (NSCLC) and BM who were not suitable for surgery or SRS.^12^

The neurological symptoms caused by BM and adverse effects of brain radiotherapy can impair patients’ QOL.^11^ It is important to consider the integration of palliative care (PC) as early as possible, as it has been shown to improve QOL and reduce overall health care utilization, such as emergency department (ED) visits and hospitalization.^13–17^ Despite this, there is limited referral to the PC unit for patients with BM.^15–18^

The aim of our retrospective study was to investigate the impact of the PC decision on the use of hospital resources and the implementation of radiotherapy at the end of life (EOL) in patients with BM.

## Patients and Methods

### Cohort selection

In this retrospective study, 104 deceased patients with BM were identified from hospital registries using the International Classification of Disease coding for BM (C79.30–79.49), including all cancer patients regardless of primary diagnosis. The identified patients had radiotherapy to the brain region at the Vaasa Central Hospital during March 2011 and March 2021 and died thereafter between May 2011 and November 2021. Although 13 patients who met a radiation oncologist but did not receive radiotherapy owing to a poor overall clinical health status were excluded from the study, 91 remained.

This study was approved by the Medical Director of the Vaasa Central Hospital. No patient interventions were performed. According to the Finnish legislation for research, no ethics committee approval was needed, as data used in the study consisted of deceased patients.

### Data collection

Data on patient demographics (age, gender, ECOG performance status) and tumor (histology) characteristics, cancer treatments (chemotherapy and radiotherapy), number of BM, timing of BM, and data on visits to the PC outpatient unit were retrospectively recorded from the medical records of the hospital. Date of deaths was collected from death certifications.

### PC decision and period

In Finland, PC is provided in primary health care and secondary/tertiary health care. In the study, a PC unit is defined as a specialized secondary hospital unit where care is provided by a PC specialist.

The PC decision, i.e., the decision to terminate curative or life prolonging anticancer treatments and focus on symptom-centered care, is made by the oncologist responsible for the care of the patient. In this study, the PC period is defined as a period of disease where curative or life-prolonging treatment can no longer be offered. The PC period covers the period from the decision to abstain cancer-specific treatment (except palliative radiotherapy) to death. If PC decision was given 30 days prior to death, it was determined as an early PC decision, whereas PC decisions made <30 days prior to death were defined as late.

## Statistical Analyses

All statistics were performed using IBM-SPSS version 28 (IBM Corp, Armonk, NY, USA). Descriptive statistics are reported as medians and ranges, numbers of incidences, and percentages. Pearson’s chi-squared test and Fisher’s exact tests were used to compare categorical variables. The overall survival (OS) was defined as the period in months from the date of BM diagnosis via radiographical imaging to the date of death. The median OS with 95% confidence interval was estimated using Kaplan–Meier analysis.

## Results

The final cohort included 91 deceased patients. The patient characteristics and treatments for BM are shown in Table 1.

**Table 1.** Patient Characteristics

Patient characteristics (*n* = 91)			
	Patients *n* (%)	No PC decision or PC decision ≤30 days before death, *n* = 59 (%)	PC decision >30 days before death, *n* = 32 (%)
Age (median 68 years)			
≤68 years	51 (56)	33 (56)	18 (56)
>68 years	40 (44)	26 (44)	14 (44)
Gender			
Female	37 (41)	21 (36)	16 (50)
Male	54 (59)	38 (64)	16 (50)
ECOG performance status			
0–1	33 (36)	22 (37)	11 (34)
2	37 (41)	21 (36)	16 (50.0)
3	21 (23)	16 (27)	5 (16)
Primary tumor type			
Other tumors	28 (31)	17 (29)	11 (34)
NSCL	22 (24)	16 (27)	6 (19)
Breast cancer	11 (12)	6 (10)	5 (16)
Melanoma	11 (12)	7 (12)	4 (13)
No histological diagnosis	11 (12)	8 (14)	3 (9)
Small cell lung cancer	8 (9)	5 (8)	3 (9)
Number of BM			
1–3	44 (48)	28 (47)	16 (50)
≥4	47 (52)	31 (53)	16 (50)
Interval from cancer diagnosis to diagnosis of BM (median 14 months)			
≤12 months	42 (46)	26 (44)	16 (50)
>12 months	49 (54)	33 (56)	16 (50)
Number of involved extracranial organs			
0	11 (12)	7 (12)	4 (13)
1	21 (23)	16 (27)	5 (16)
2	26 (29)	14 (24)	12 (37)
3 or more	33 (36)	22 (37)	11 (34)
WBRT regimen			
20 Gy in 5 fractions	19 (21)	12 (20)	7 (22)
30 Gy in 10 fractions	66 (72)	43 (73)	23 (72)
Stereotactic radiotherapy	6 (7)	4 (7)	2 (6)
Radiotherapy for recurrent metastases	6 (7)	3 (5)	3 (9)
Systemic treatment after radiotherapy	33 (36)	21 (35)	12 (37)
Median time from diagnosis of BM to start of radiotherapy	17 days	17 days (5–167 days)	19 days (1–182 days)
Median time from start of radiotherapy to death	75 days	54 (range 17–1002 days)	236 (range 36–1862 days)
PC decision	79 (87)	47 (80)	32 (100)
Median time from PC decision to death	18 days	10 (range 1–27 days)	79 (range 31–621 days)
Median OS after diagnosis of BM	112 days	75 days (29–1047 days)	255 days (44–1864 days)

BM, brain metastases; NSCL, non-small cell lung cancer; OS, overall survival; PC, palliative care; WBRT, whole brain radiation therapy.

The mean age of the patients was 67 years (range 23–91). Lung cancer was the most common tumor type (33%) followed by breast cancer (12%). Altogether, 52% of the patients had multiple BM (≥4 metastases) and 88% had extracranial metastases simultaneously. The median interval for the detection of BM after cancer diagnosis was 14 months (range 0 months to 23 years 7 months).

**FIG. 1.**
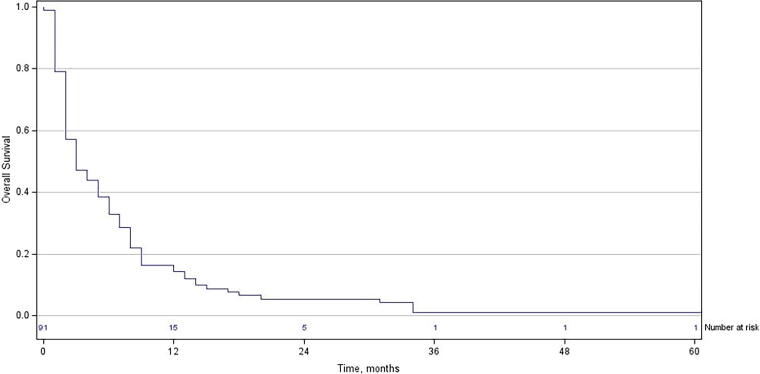
Kaplan–Meier curve for OS in the patient population. OS, overall survival.

**FIG. 2.**
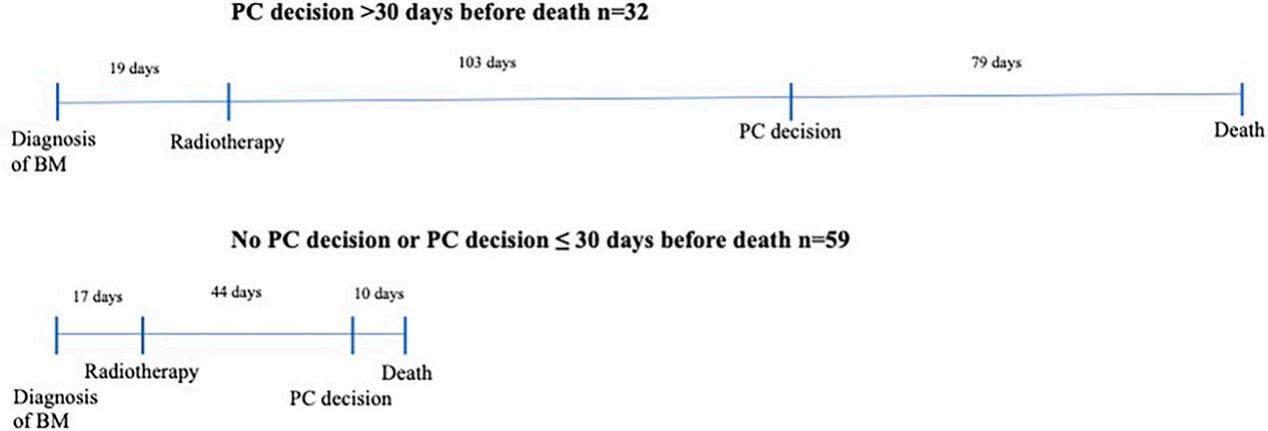
Time from diagnosis of BM to radiotherapy and death, comparing groups by timing of PC decision. All values are medians. BM, brain metastases; PC, palliative care.

Median OS from diagnosis of BM was 3.7 months (range 1 month to 5 years 2 months) and the survival rates at 6 and 12 months were 38% and 16%, respectively (Fig. 1). Median survival by tumor type was 6.3 months in melanoma, 5.5 months in breast cancer, 3.9 months in NSCLC, and 3.8 months in small cell lung cancer. The disease behaved aggressively in 65% of patients, and a median of 54 days of days of life remained after radiotherapy. Timelines are shown in Figure 2.

Patients were treated mainly with WBRT 30 Gy in 3 Gy daily fractions (72%) and only a few with SRT (7%). Twenty-nine patients (32%) had radiotherapy during the last 30 days of life. Almost all patients (96%) completed the palliative radiotherapy. The reasons for discontinuing radiotherapy were owing to deterioration in overall clinical health status. Six patients (6%) received repeated radiotherapy to the brain area, most of whom received SRT (83%), and 33 patients (36%) continued systemic treatment after radiotherapy to the brain area.

All of the 15 patients (16%) that underwent surgical resection of the metastases or had a biopsy taken before radiotherapy received WBRT, of which three received an additional 3–4 fraction booster to the resection/tumor site. The median survival time for these patients was 9.8 months, which is higher than in the entire cohort.

Almost all (98%) patients had neurological symptoms at the time of detection of BM and initiation of radiotherapy. The most common symptoms were seizure, dizziness, and paralysis. Almost every patient (93%) had dexamethasone treatment during radiotherapy.

## PC Decision

PC decision was defined for 79 (87%) patients of which only 7 (8%) had the PC decision made before the implementation of RT. The median survival time after the PC decision was 18 days (range 0–621). Thirty-two patients (35%) had a PC decision made more than 30 days prior to death. Patients who had an early PC decision (>30 days before death) were more likely to visit the PC outpatient unit.

One-fifth of the patients (19%) visited or contacted the PC unit. Median survival after PC consultation was 44 days. Most of the study population (78%) did have a do not resuscitate order.

## Use of Hospital Services

The majority of patients (67%) had ED visits after receiving radiotherapy and were hospitalized (66%) to the central hospital. In the last 30 days of life, 44 patients (48%) had an ED visit and almost all these patients (38 patients, 86%) were hospitalized to the central hospital. The average length of the stay in the hospital was 6.7 days (range 2–20). Nineteen (50%) of the patients who were hospitalized at EOL died at the central hospital.

**FIG. 3.**
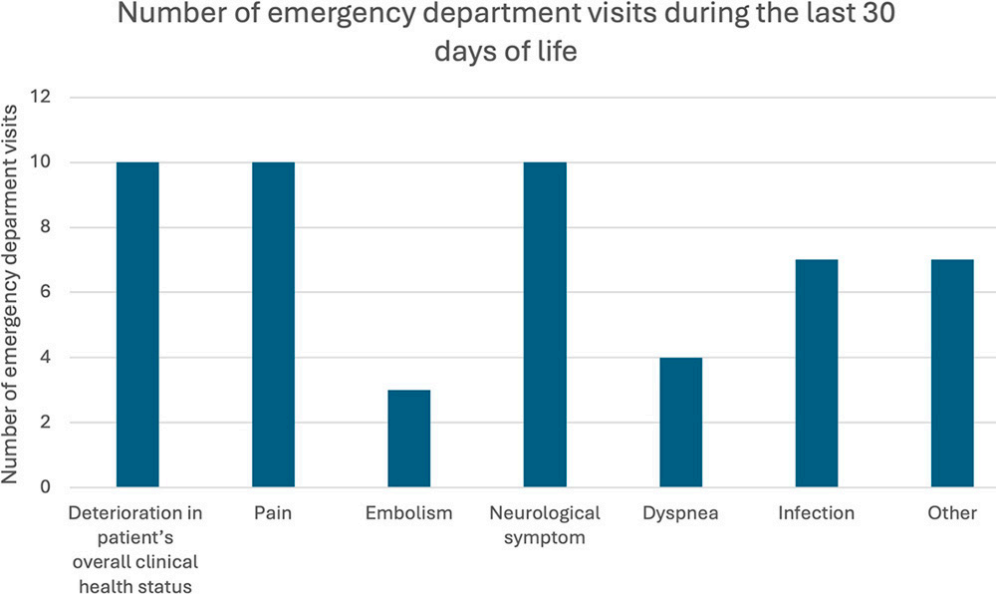
Reasons for emergency department visits during the last 30 days of life.

Reasons for ED visits during the last 30 days of life are shown in Figure 3. Altogether, 20% of the patients visited the ED owing to neurological symptoms, which seem to be related to the intracranial process. Symptoms caused by extracranial disease progression explained 47% of the visits (deterioration in overall clinical health status, pain, and dyspnea).

Fewer patients with early PC decision (>30 days before death) were hospitalized (22% vs. 53%; *p* value 0.005) and died during the hospitalization period (9% vs. 27%; *p* value 0.047) at EOL compared with patients with no or late (≤30 days before death) PC decision. The effect of a PC decision timing on the use of hospital services is shown in Table 2.

**Table 2.** The Effect of a Palliative Care Decision Timing on the Use of Hospital Services and the Last Radiotherapy

	No PC decision or PC decision ≤30 days before death, *n* = 59 (%)	PC decision >30 days before death, *n* = 32 (%)	*p* value
Number of the patients who used central hospital services in the last 30 days of life			
ED visits	31 (53)	13 (41)	0.277
Hospitalization at central hospital	31 (53)	7 (22)	0.005
Died during the hospitalization period	16 (27)	3 (9)	0.047
Last radiotherapy			
30 days before death	27 (46)	2 (6)	<0.001
14 days before death	9 (15)	1 (3)	0.077
PC unit visits	5 (8)	12 (38)	<0.001

ED, emergency department.

## Discussion

In this retrospective single-institution cohort of patients with BM treated in the radiotherapy department, we demonstrated an aggressive treatment strategy despite poor prognoses. Every third (32%) patient was treated with radiotherapy during the last month of life. The PC decision i.e., termination of life-prolonging cancer treatments and focusing on symptom-centered PC was made late in life and very few patients were referred to PC unit leading to increased number of ED visits and hospitalizations at the EOL.

The median OS of the present cohort was shorter than reported in previous studies, 3.7 months after diagnosis of BM.^6,10,11,20^ However, one-third of the study patients (31%) survived over 7 months after starting radiotherapy. Füreder et al.^6^ reported a median survival time of six months after diagnosis of BM in patients with known primary tumor and 8 months in patients with BM as the first sign of advanced cancer. In another population-based study, median OS was 5 months among all cancer patients with BM at diagnosis.^10^ Compared with the study by Füreder et al.,^6^ most of the patients in our study had poor performance status, multiple BM, and metastases in several extracranial organs at the time of BM diagnosis, indicating aggressive treatment strategy despite signs of poor prognoses. In addition, compared with a recently published meta-analysis of survival in patients with BM, our study population had significantly shorter survival (weighted median OS for patients with stable intracranial disease was 17.9 months and 8 months for patients with progressive intracranial disease).^26^ Furthermore, only patients who received radiotherapy were included in the present study and they were treated between 2011 and 2021, so treatment practices may differ from today.

Another sign of an aggressive treatment strategy in this present cohort was a large proportion of patients (72%) treated with long-term WBRT treatment (30 Gy 3 Gy fractions) compared with a population-based cohort of 2698 BM patients treated in Australia, of whom 51% received short-term WBRT treatment (≤6 fractions).^19^ The use of SRS in the present study was comparable with Australian and NSCLC studies (7% vs. 10% vs. 14%, respectively).^19,20^ Furthermore, 32% of the patients in the present study received radiotherapy during the last 30 days of life. Ryoo et al.^20^ reported that 23% patients with NSCLC who received brain radiotherapy died within 30 days of last treatment. According to Astro’s guidelines, refraining from radiotherapy is recommended if the prognosis of patients with BM is assessed as poor (ECOG performance status 3–4, systemic disease with poor treatment options, and symptoms of BM controlled with cortisone).^21^ The QUARTZ randomized clinical trial showed that NSCLC patients with BM who were not suitable for resection or SRS, did not benefit from WBRT, and no difference in neither OS nor QOL was seen between those treated with radiotherapy or supportive care alone.^12^ For patients with BM, it would therefore be useful to consider whether radiotherapy (WBRT or SRS) should be given or whether high-quality symptomatic care should be provided instead.

According to the international recommendation, patients with BM should be systematically referred to the PC unit for early integration of PC approaches to enhance cancer patients’ QOL and to provide support.^13,14,24,25^ In line with previous studies, referral of patients with BM to the PC unit was limited in our study.^15,18^ Only 19% of the patients were referred to the PC unit compared with the study by McDermott et al.^18^ where 48% (49/103) of NSCLC patients with BM had a PC consultation. In both studies, however, referrals were made late, with a median 1.6 months before death in the McDermott study as compared with our 1.5 months.

For patients with better prognoses, PC decisions were made well in advance (median 79 days before death), and they were referred to the PC unit more frequently (38% of them). In contrast, patients with more aggressive disease were not identified, PC decisions were made late, and only 9% of them were referred to a PC unit. They were also more likely to receive radiotherapy in the last month of life compared with patients for whom the PC decision was made in advance (46% vs. 6%).

In our study group, 67% of patients visited the ED after radiotherapy. In the last month of life, 48% of patients visited the ED, and most of them (86%) were hospitalized. Half of these patients died during their hospitalization in a central hospital. Patients who had an early PC decision had fewer hospital stays (*p* value 0.005) and death during hospitalization period (*p* value 0.047) compared with patients for whom a PC decision was not made or made late. Although there was no statistically significant decrease in ED visits for patients with an early PC decision, there was still a reduction in hospital stays and hospital deaths. In a randomly chosen cohort of 992 Finnish cancer patients, early PC decision (>30 days before death) decreased the number of ED visits and inpatient days in a secondary/tertiary care hospital.^22^ In another study, Blackhall et al.^23^ showed that referral to outpatient PC within 3 months of death reduced hospitalizations at EOL, patients were less likely to die at hospital, had increased hospice utilization, and decreased costs of care.

Thus, BM should be a trigger for referral to the PC unit and consequently for consideration of discontinuation of oncological treatments and withholding radiotherapy if there are signs of poor prognosis. If the PC decision is delayed, the risk of ending up in the ED and dying during hospitalization increases, which is not consistent with quality EOL care.

There are some limitations to this study. The study was conducted as a single-center study and was retrospective in design. QOL data were not available. The size of the study population was small, and it was heterogeneous. Another limitation is that the study only included patients who received radiotherapy and did not include all patients with BM. This strengthens our findings even more, however, as patients with limited life expectancy were more likely not to be referred to the radiotherapy department, the prognoses in our cohort was very poor. The age of the data is another limitation, considering the advances in anticancer therapies in recent years. The strength of the study is its real-life nature, illustrating clinical practice in real life. In addition, the follow-up period was sufficient; patients were followed from BM diagnosis until death.

## Conclusions

BM are a sign of a poor prognosis in most patients and should be an indicator for referral to a PC unit. It is important to identify patients for whom the disease behaves aggressively and in whom BM is a sign of impending death. Their EOL care should be consequently organized appropriately, thus preventing the administration of radiotherapy at EOL and reducing inappropriate health care utilization.

## Ethical Approval and Consent to Participate

The present study is a retrospective register study based on hospital registry data of deceased patients from years 2011 to 2021. No patient interventions were performed. Study was performed with the permission of the authorities of Vaasa Central Hospital. Data were anonymized before their use. We confirm that all methods were carried out in accordance with relevant guidelines and regulations. According to the Finnish law, the legislation does not mandate any Ethics Committee approval. The study was done on deceased patients. There was no need for informed consents.

## Data Availability

The datasets analyzed during the current study are available from the corresponding author on reasonable request.

## Abbreviations Used

BM: brain metastases
ED: emergency department
EOL: end of life
NSCLC: nonsmall cell lung cancer
OS: overall survival
PC: palliative care
QOL: quality of life
QUARTZ: Quality of Life after Treatment for Brain Metastases
SRS/SRT: stereotactic radiotherapy
WBRT: whole brain radiation therapy
